# Generation of *APOE* knock-down SK-N-SH human neuroblastoma cells using CRISPR/Cas9: a novel cellular model relevant to Alzheimer’s disease research

**DOI:** 10.1042/BSR20204243

**Published:** 2021-02-19

**Authors:** Sonia Sanz Muñoz, Brett Garner, Lezanne Ooi

**Affiliations:** 1 Illawarra Health and Medical Research Institute, Wollongong, NSW 2522, Australia; 2 School of Chemistry and Molecular Bioscience, University of Wollongong, Wollongong, NSW 2522, Australia

**Keywords:** Alzheimer's disease, ApoE, apoliprotein E, HtrA1, neuritogenesis

## Abstract

*APOE* ε4 is the major genetic risk factor for Alzheimer’s disease (AD). A precise role for apolipoprotein E (apoE) in the pathogenesis of the disease remains unclear in part due to its expression in multiple cell types of the brain. *APOE* is highly expressed in astrocytes and microglia, however its expression can also be induced in neurons under various conditions. The neuron-like cell line SK-N-SH is a useful model in the study of the cellular and molecular effects of apoE as it can be differentiated with retinoic acid to express and secrete high levels of apoE and it also shows the same apoE fragmentation patterns observed in the human brain. We previously found that apoE is cleaved into a 25-kDa fragment by high temperature-requirement serine protease A1 (HtrA1) in SK-N-SH cells. To further understand the endogenous functions of apoE, we used CRISPR/Cas9 to generate SK-N-SH cell lines with *APOE* expression knocked-down (KD). *APOE* KD cells showed lower *APOE* and *HTRA1* expression than parental SK-N-SH cells but no overt differences in neuritogenesis or cell proliferation compared with the CRISPR/Cas9 control cells. This research shows that the loss of apoE and HtrA1 has a negligible effect on neuritogenesis and cell survival in SK-N-SH neuron-like cells.

## Introduction

Alzheimer’s disease (AD) is the most common neurodegenerative disorder and the presence of the *APOE ε*4 allele is the major genetic risk factor for the disease [1]. Apolipoprotein E (apoE) is widely expressed in the human body; in the brain, it is expressed by astrocytes and microglia, and also by neurons under certain circumstances [2,3].

ApoE is fragmented in the human brain in an isoform-specific manner [4] generating truncated fragments (as reviewed in [5]). Specifically, the apoE 25-kDa N-terminal fragment (apoE 25) is present at higher levels in apoE3 compared with apoE4 brains [4], and the proteolysis of apoE *in vitro* mediated by the high temperature-requirement serine protease A1 (HtrA1) enzyme is allele selective, with increased fragmentation of apoE3 compared with apoE4 [6].

The SK-N-SH neuroblastoma cell line has been previously used to test neuroprotective compounds against some AD-relevant features, such as amyloid β [7,8], oxidative stress [9,10] and apoptosis [11,12]. Moreover, SK-N-SH cells have also been used to study apoE fragmentation [13–15], where it was shown that the apoE 25-kDa fragment is generated by HtrA1 [16]. Despite this, the role of apoE in SK-N-SH cells remains unclear as at least two distinct cell populations are present after differentiation: the ‘neuroblast-like’ (N-type) and ‘epithelial-like’ or substrate-adherent (S-type) cells together with an intermediate state (I-type) with a mixed expression pattern [17–19]. SH-SY5Y cells, created from a subclone of SK-N-SH cells, contain only the N-type cells [20–22] but with subpopulation clusters of specific surface markers [23]. The SK-N-SH neuroblastoma cell line has been used as a model to study apoE fragmentation, including the apoE 25-kDa fragment that is generated by HtrA1 [16]. Inhibition of HtrA1 activity or *HTRA1* knockdown in SK-N-SH cells reduced apoE 25, whilst incubation of recombinant apoE 25 in SH-SY5Y cells promoted neuritogenesis [16].

The *APOE* ε3/3 background of the SK-N-SH cells limits the model when trying to understand the apoE4 isoform and its fragmentation process. The aim of this research was to knock-down *APOE* expression using CRISPR/Cas9 genome editing to provide a cellular model for investigating the role of apoE and its effect on HtrA1 in SK-N-SH cells.

## Materials and methods

### Cell culture and neuronal differentiation of neuroblastoma cells

Human SK-N-SH neuroblastoma cells were obtained from American Type Culture Collection (ATCC, Manassas, VA, U.S.A.; Cat# HTB11). Cell line identity was confirmed by short tandem repeat (STR) profiling at the Garvan Molecular Institute (Darlinghurst, Australia), with results matching ATCC HTB-11. Analysis for potential mycoplasma contamination of the parental SK-N-SH and the clones tested negative. SK-N-SH cells were cultured in Dulbecco’s modified Eagle’s medium (DMEM)/F12 (v/v) (1:1) (Invitrogen, U.S.A.) supplemented with 10% (v/v) Foetal Bovine Serum (FBS) (Interpath Services, Australia) and 100 IU/ml Penicillin, and 100 µg/ml Streptomycin (Life Technologies, U.S.A.) at 37°C and 5% CO_2_. The medium was changed every 2–3 days and cells were passaged when 80% confluence was reached. Cells were incubated with Trypsin (0.05% Trypsin-Ethylenediaminetetraacetic acid (EDTA)) (Life Technologies) for 3–5 min at 37°C and stopped using DMEM/F12 with 10% (v/v) FBS, after which cells were centrifuged at 800×***g*** for 5 min and split 1:10.

SK-N-SH cells were differentiated into a neuronal phenotype with the addition of 10 µM all-*trans* retinoic acid (ATRA). Cells were plated at the density specified for each experiment using DMEM/F12 with 5% (v/v) FBS unless otherwise specified. Cells were grown for 24 h before ATRA treatment started, and full media changes were performed every 3 days until day 6 or 9 as specified in each experiment. Live cell imaging of the cultures, cell confluence and neurite outgrowth measurements over time were performed using the Incucyte Zoom Imaging System (Essen Bioscience, U.S.A.).

### CRISPR lentivirus design

**Figure 1:**
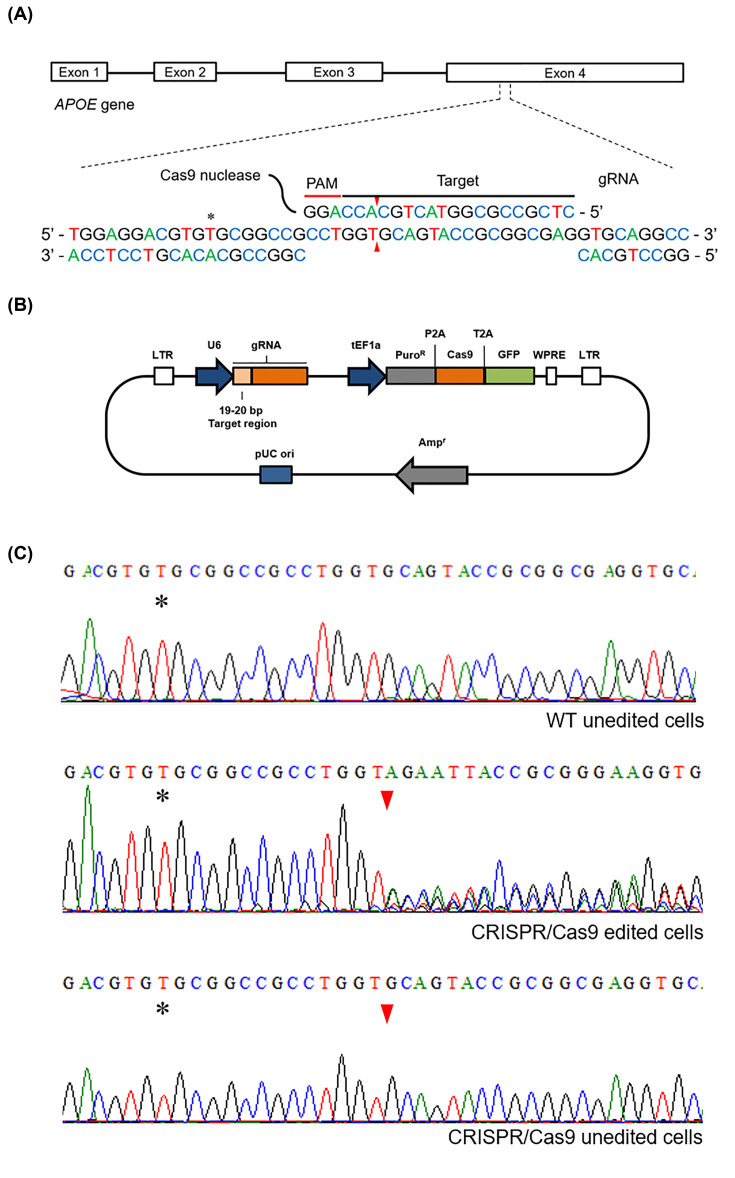
CRISPR/Cas9 design, plasmid and sequencing (**A**) Schematic diagram of the gRNA1 targeting exon 4 of the *APOE* gene: the four exons of the *APOE* gene are shown, with the gRNA1 targeting a specific region of exon 4. The gRNA contains the protospacer adjacent motif (PAM) sequence linked to the Cas9 nuclease that will induce a double strand break (DSB) 3 bp upstream of the PAM, represented with red triangles. The asterisk (*) indicates the nucleotide that determines the *APOE ε*3/3 background. (**B**) The all-in-one lentivirus CRISPR vector contains the specific gRNA under the U6 promoter and the puromycin resistance cassette (Puro^R^), Cas9 nuclease and green fluorescent protein (GFP) tag under the translation elongation factor 1 α (tEF1a) promoter. (**C**) Sequencing electropherograms of SK-N-SH cells targeting the region of interest of the *APOE* gene showing WT unedited cells, a double peak electropherogram from the predicted DSB region in CRISPR/Cas9 edited cells and a normal sequence in the *APOE* region in CRISPR/Cas9-edited cells. Asterisk indicates the nucleotide that determines the *APOE ε*3/3 background and red triangle indicates the predicted cut site of the Cas9 nuclease.

The CRISPR lentivirus plasmid with guide RNA (gRNA) sequence 5′-CTCGCCGCGGTACTGCACCAGG-3′ was designed by Sigma–Aldrich to target exon 4 of the *APOE* gene as shown in Figure 1A. The *in silico* on- and off-target score screening of gRNA1 was performed using the online platform Benchling [24,25].

The plasmid, as shown in Figure 1B, contained the gRNA under U6 promotor expression, puromycin resistance cassette, Cas9 protein expression cassette and a GFP expression cassette under the expression of the translation elongation factor 1 α (tEF1a) promoter. It also contained an ampicillin resistance cassette for cloning, enzyme restriction cutting sites, flanking long terminal repeats (LTRs) to facilitate the integration into the host genome and a Woodchuck hepatitis virus (WHP) post-transcriptional regulatory element (WPRE) to enhance the expression of the delivered genes.

### Puromycin killing curve

SK-N-SH cells were plated at 16,000 cells/well on a 96-well plate before treatment with puromycin (Sigma–Aldrich). The following puromycin concentrations were added in triplicate 24 h after seeding: 10, 5, 2.5, 1.25, 0.625, 0.3125 and 0 µg/ml as control. Cell death was tracked using the Incucyte Zoom System every 2 h for a period of 6 days. After 5 days, all cells from the 2.5 µg/ml or higher concentration were dead, and only <5% of the 1.25 µg/ml had survived. Therefore, the concentration of 2 µg/ml was chosen to perform the antibiotic selection of cells after testing that all the cells died in a 5-day period.

### CRISPR lentiviral transduction

SK-N-SH neuroblastoma cells were plated at 100,000 cells/well in a six-well plate. Base media (DMEM/F12 media with 10% (v/v) FBS, 100 IU/ml Penicillin and 100 µg/ml Streptomycin) was supplemented with 8 µg/ml of polybrene to enhance the transduction efficiency. Serial dilutions of the CRISPR lentivirus over a range of 10^−2^ to 10^−6^ dilution were performed as recommended by the manufacturer together with a no-lentivirus control well. Full media change with base media was performed 24 h after to remove the lentiviral particles from the culture. Antibiotic selection using 2 µg/ml puromycin started 48 h after removal of the lentiviral particles until all the cells in the control well were dead. Transduced cells were kept in culture without antibiotic to allow their expansion; cells were passaged into a six-well plate, allowing them to grow from individual cells into groups of ten colonies and then they were manually picked for monoclonal selection. Clones were cultured in 96-well plates and expanded into 24- and 12-well plates when they reached 80% confluency until there were enough cells to extract gDNA, harvest protein and make stocks.

### DNA extraction and sequencing

Genomic DNA was extracted using the ISOLATE II Genomic DNA Kit (Bioline). DNA concentration and purity were determined using the Nanodrop 2000C instrument. PCR was performed by mixing 100–200 ng DNA with 12.5 µl MyTaq™ HS DNA Polymerase, 10 µM of primers *APOE* F (5′-CTACAAATCGGAACTGGAGGAAC-3′) and *APOE* R (5′- ACCTGCTCCTTCACCTCGTC-3′) and water up to 25 µl reaction. PCR was amplified in the MyCycler™ Thermal Cycler System (Bio-Rad, U.S.A.) using the following program: initial denaturation at 95°C for 1 min, followed by 35 cycles of 15 s at 95°C for denaturation, 15 s at 66°C and 10 s at 72°C for extension and final extension at 72°C for 5 min.

Electrophoresis was performed using 4 µl of each sample mixed with GelRed Nucleic Acid Gel Stain and run on 1.5% agarose gel at 100 V for 30 min run along with Hyperladder 100 bp and visualised on Gel Logic 2200 Pro Imaging System. After confirming the specific DNA amplification of 489 bp, PCR products were purified using a modified protocol of Pure Link Quick Gel Extraction kit (Invitrogen): 16 µl PCR product were mixed with 20 µl water and 120 µl buffer L3, transferred into the columns and centrifuged at 12000×***g*** for 1 min. Flow through was discarded, 600 µl wash buffer were added and centrifuged at 12000×***g*** for 1 min. Flow through was discarded and columns were centrifuged at maximum speed for 2 min to remove any buffer. After transferring the column into a 1.5 ml microcentrifuge tube, 20 µl of elution buffer was added and samples were centrifuged at 12000×***g*** for 1 min to collect the purified PCR product.

Sequencing reactions were performed by mixing 2 µl of 5× Sequencing buffer, 1 µl ABI BigDye Terminator v3.1 Ready Reaction Mix (Applied Biosystems, U.S.A.), 1 µl Primer F or R (2 µM), 4 µl water and 2 µl of purified PCR product. Samples were run on the MyCycler™ Thermal Cycler System following the program: 96°C for 30 s followed by 35 cycles of 96°C for 10 s, 66°C for 5 s and 60°C for 4 min.

Ethanol precipitation was performed by mixing 50 µl 100% ethanol, 10 µl water, 2 µl 125 mM EDTA pH 8.0, 2 µl 3 M sodium acetate pH 5.2 and 10 µl sequencing reaction. The samples were incubated at 22°C in the dark for 20 min after mixing before they were centrifuged at 16000×***g*** for 20 min at 22°C. The supernatant was removed, the pellet was rinsed with 180 µl ice-cold 70% ethanol and the samples were centrifuged for 20 min at 4°C. The supernatant was removed and the samples were dried in the dark before being processed.

Samples were run in the 3500×L Genetic Analyzer (Applied Biosystems). SeqA6 Software was used to convert raw files into .ab1 files and electropherograms were analysed using BioEdit software.

### mRNA extraction and qRT-PCR

Cell samples for mRNA extraction were washed twice with 1× Phosphate Buffered Saline (PBS) before they were harvested in TriSURE (Bioline). Samples were kept at −80°C until mRNA was extracted, following the manufacturer’s instructions. The mRNA concentration was measured using the NanoDrop 2000C and purity was assessed based on the A_260/230_ and A_260/280_ ratios. DNAse I treatment was performed using Turbo DNA-free kit (Invitrogen) following the manufacturer’s instructions. The mRNA was purified by ethanol precipitation: mRNA was diluted up to 200 µl RNAse-free water and mixed with 20 µl of 3 M Na acetate and 220 µl isopropanol. After vortexing, samples were stored at −80°C for 1 h and then centrifuged at 16000×***g*** for 20 min at 4°C. The supernatant was discarded, and the pellet was rinsed with 70% ethanol before being centrifuged again at maximum speed for 2 min. The supernatant was removed, and the pellet was dried before it was resuspended in RNAse-free water to yield a final concentration between 100 and 500 ng/µl. Purity and concentration of the isolated mRNA were measured using the Nanodrop 2000C.

Up to 5 µg of mRNA were reverse transcribed to generate complementary DNA (cDNA) using Tetro cDNA Synthesis kit (Bioline). The protocol was followed as per manufacturer’s instructions using Oligo (dT)_18_ and incubating samples for 30 min at 45°C followed by 30 min at 85°C in MyCycler Thermal Cycler. The samples were stored at −20°C until use.

Each cDNA sample was run in duplicates or triplicates using between 25 and 50 ng cDNA per reaction. A ‘no reverse transcriptase’ RNA control and ‘no template’ control were also run as negative controls. The qRT-PCR machine LightCycler 480 (Roche, Switzerland) or RotorGene 3000 (Qiagen, Germany) were used following the program: denaturation at 95°C for 2 min, followed by 35 or 40 cycles of denaturation at 95°C for 10 s, annealing at specific primer Tm for 10 s and extension at 72°C for 10 s. The melting step started at 95°C for 5 s followed by ramp from 65 to 97°C at 0.1°C/s before cooling. Primer sequences are shown in Table 1. All *C*_t_ values were in the range between cycles 20 and 32, for *APOE* and *HTRA1*, and between cycles 15 and 22 for the housekeepers, *GAPDH* and *HPRT1*.

**Table 1:** List of primers used for qRT-PCR

Target	Sequence (5′–3′)	Tm	Amplicon size
*APOE* (1)	F: CCAATCACAGGCAGGAAGAT	62°C	253 bp
	R: GCAGGTAATCCCAAAAGCGAC		
*APOE* (2)	F: GTCGCTTTTGGGATTACCTGC	62°C	151 bp
	R: CCGGGGTCAGTTGTTCCTC		
*APOE* (3)	F: GAGGTGAAGGAGCAGGTGG	62°C	218 bp
	R: TTCGGCGTTCAGTGATTGTC		
*APOE* (4)	F: GACAATCACTGAACGCCGAAG	62°C	192 bp
	R: TGCGTGAAACTTGGTGAATCTT		
*HTRA1*	F: GACTACATCCAGACCGACGC	62°C	246 bp
	R: TTTGGCTTTGCTGGACGTGA		
*GAPDH*	F: GAGCACAAGAGGAAGAGAGAGACCC	58–62°C	214 bp
	R: GTTGAGCACAGGGTACTTTATTGATGGTACATG		
*HPRT1*	F: TGACACTGGCAAAACAATGCA	58–62°C	150 bp
	R: GGTCCTTTTCACCAGCAAGCT		

Raw data from LightCycler 480 or RotorGene 3000 instruments were extracted and analysed using LinRegPCR [26] to obtain the individual efficiency of each reaction. Data analysis was performed using Excel following comparative *C*_T_ (ΔΔ*C*_t_) method using the efficiency obtained for each set of primers. GraphPad Prism 7 was used to generate graphs and perform statistical analysis.

### Protein extraction and Western blot

Conditioned medium from cell culture was collected to analyse extracellular proteins released into the media. The conditioned medium was collected directly from the plate, centrifuged at 10000×***g*** for 5 min to remove cell debris and dead cells, transferred into a 1.5 ml microcentrifuge tube and stored at −80°C until use.

Intracellular protein was harvested in RIPA buffer (50 mM Tris HCl pH 7.4, 1% sodium deoxycholate, 150 mM NaCl, 1 mM EDTA, 1% Triton-X and 0.1% SDS in Milli-Q water) mixed with 1× protease inhibitor cocktail (#P8340, Sigma–Aldrich, U.S.A.) and 0.1 M phosphatase inhibitor sodium orthovanadate. Once conditioned medium was collected, cells were rinsed with 1× PBS and incubated with 100 µl RIPA buffer per well of a 24-well plate for 20 min on ice. Cell lysate was vortexed for 10 s and centrifuged at 10000×***g*** for 5 min. The supernatant was transferred into a 1.5 ml microcentrifuge tube avoiding the small debris pellet at the bottom of the tube. The cell lysate was stored at −80°C until use.

Total protein quantification was performed using the DC Protein Assay (Bio-Rad), following the manufacturer’s instructions. Absorbance was measured at 750 nm using a Spectramax Plus Plate Reader (Molecular Devices, U.S.A.). The sample concentration was calculated based on a standard curve generated using Bovine Serum Albumin (BSA) (Sigma–Aldrich) diluted in RIPA buffer at concentrations of 0, 200, 400, 600, 800 and 1200 µg/ml.

Samples were mixed with 5× Laemmli Sample Buffer (Bio-Rad) and proteins were denatured for 5 min at 95°C. Samples were loaded into SDS/PAGE gels along with the Precision Plus Protein Dual Color (Bio-Rad) molecular weight marker and separated by electrophoresis at 120 V for 80 min. Protein samples were transferred to 0.45-µm nitrocellulose membranes at 100 V for 35 min using ice-cold 1× Western blot transfer buffer. Successful transfer and equal sample loading were determined by staining the membranes with Ponceau S Solution Bioreagent (Sigma–Aldrich) for 2 min and the excess stain was washed off with distilled water. The membranes were scanned on Epson Perfection 3170 Photo (Epson, Japan), then washed off for 5 min with 1× PBS-0.1% (v/v) Tween 20 (PBS-T) followed by incubation for 5 min with boiling 1× PBS and another 5 min with 1× PBS-T. Blocking for 1 h with 5% (w/v) milk in 1× PBS was performed before incubation with primary antibodies made up in 5% (w/v) milk in 1× PBS and 0.001% (v/v) sodium azide. Antibody details are shown in Table 2. Primary antibodies were incubated at 4°C for 16 h, followed by three washes with 1× PBS-T for 10 min each. Secondary antibodies conjugated with HRP were made up in 5% (w/v) milk in 1× PBS-T and incubated for 2 h at 22°C, followed by 3 washes of 1× PBS-T for 10 min each. Membranes were incubated for 2 min with Pierce ECL Plus Western Blotting Substrate (Thermo Fisher Scientific). Protein expression was detected by chemiluminescence on Amersham 6000RGB (GE Healthcare Life Sciences) instrument or after exposing the membranes to X-ray film developed using the AGFA-CP1000 Film Processor.

**Table 2:** List of antibodies used for Western blot

Antibody	Raised in	Dilution	Company Cat #
Anti-apoE	Goat pAb	1:10000	Millipore Cat #178479
Anti-GAPDH	Rabbit pAb	1:10000	Osenses Cat #OSG00032W
Anti-Goat HRP	Donkey pAb	1:5000	Jackson ImmunoResearch Labs Cat #705-035-147
Anti-Rabbit HRP	Donkey pAb	1:5000	Jackson ImmunoResearch Labs Cat #711-035-152

LI-COR Image Studio was used for quantification of Western blot bands, Excel was used for data analysis, and GraphPad Prism 7 was used to generate graphs and perform statistical analysis.

## Results

The SK-N-SH neuroblastoma cell line is a useful model to study apoE fragmentation (as reviewed in [5]). To further investigate the role of apoE, the CRISPR/Cas9 technique was chosen to generate *APOE* knock-down SK-N-SH cell lines to compare with the parental *APOE ɛ*3/3 SK-N-SH cells.

To predict the gRNA specificity, an *in silico* on- and off-target screening of the gRNA sequence was performed. The prediction of the on- and off-target specificity scores range from 0 to 100, where higher values indicate a more specific sequence [24,25]. The gRNA obtained an on-target score of 69.4 and an off-target score of 83.2. Within the off-target sites, *TMEM175* gene had a score of 1.43 and 4 mismatches in the sequence and a non-coding region in chromosome 1 obtained a score of 1.41 and 3 mismatches, compared with the *APOE* gene having a score of 100 and no mismatches in the sequence. This indicated a good specificity of the selected gRNA to proceed.

The pmax-GFP vector was used as a control for positive lipofectamine transfection, giving a 21% transfection efficiency after 24 h, and puromycin selection was performed until 100% of the cells in the control condition were dead (data not shown).

When comparing the DNA sequences with the SK-N-SH parental cells (Figure 1C, WT unedited cells), 13 of the clones analysed showed a double peak in the electropherogram starting from the nucleotide where the double-strand break (DSB) was predicted (Figure 1C, CRISPR/Cas9 edited cells) and others retained the normal *APOE* sequence (Figure 1C, CRISPR/Cas9 unedited cells).

**Figure 2:**
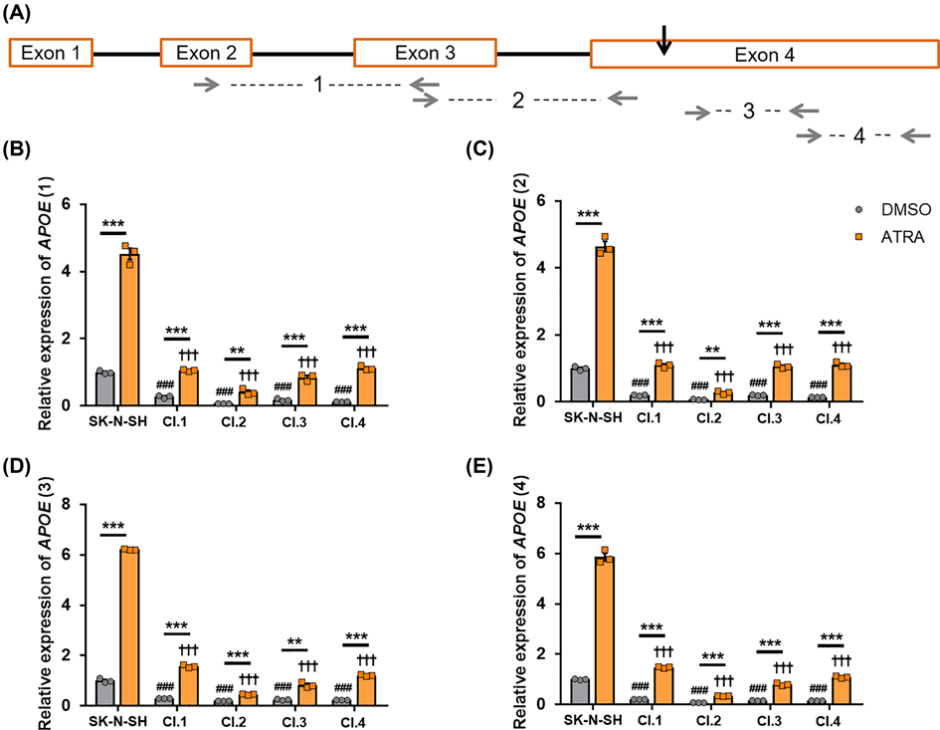
Selected clones show lower *APOE* expression than parental SK-N-SH cells SK-N-SH cells and selected clones 1–4 were cultured with 10 µM ATRA or DMSO control for 6 days. (**A**) Schematic representation of the *APOE* gene showing the four exons. Black arrow shows the targeted DSB region with the lentivirus containing gRNA1. (**B**–**E**) The mRNA expression of *APOE* using four sets of primers targeting the regions showed with grey arrows in (A) was normalised to the housekeeper genes *GAPDH* and *HPRT1* and SK-N-SH. The expression levels in DMSO vehicle control conditions was used as 1 for reference. Data in (B–E) are derived from one representative experiment that was performed in triplicate. Individual data points are shown. Histogram bars represent mean values and error bars represent S.E.M. Graphs show **P<0.01 and ***P<0.001, compared with each DMSO condition by two-tailed *t* test; ^###^*P*<0.001 compared with the SK-N-SH DMSO condition and ^†††^*P*<0.001 compared with the SK-N-SH ATRA condition by one-way ANOVA with Dunnett’s *post-hoc* analysis.

Four of the clones showing alterations in the *APOE* sequence (clones 1, 2, 3 and 4) were further characterised to confirm an *APOE* knock-down (KD) after the DSB. After neuronal differentiation with 10 µM ATRA, the parental SK-N-SH cells up-regulate apoE expression [16]. To characterise the levels of apoE expression, the parental SK-N-SH cells and the four selected clones with potential *APOE* KD were analysed via qPCR after ATRA treatment (or DMSO as a vehicle control) for 6 days (Figure 2). As the gRNA was directed to induce a break in the exon 4 of the *APOE* gene (black arrow in Figure 2), the sets of primers *APOE* 1 and 2 were designed to target the gene region before the DSB and the primers *APOE* 3 and 4 after the DSB (Figure 2A). The SK-N-SH cells and the four selected clones showed an increase in *APOE* expression after ATRA treatment when compared with each DMSO control (Figure 2B–E). However, the *APOE* expression of the four clones was significantly lower when compared with the respective condition of the parental SK-N-SH cells (Figure 2B–E). All the selected clones showed a reduction of more than 70% when compared with the parental SK-N-SH cells under the respective conditions. Clone 2 reached a reduction of 94% after ATRA treatment when compared with the SK-N-SH parental cell lines. The expression pattern of the selected clones followed the same trend in the primers targeting the sequence before and after the DSB (Figure 2B–E).

**Figure 3:**
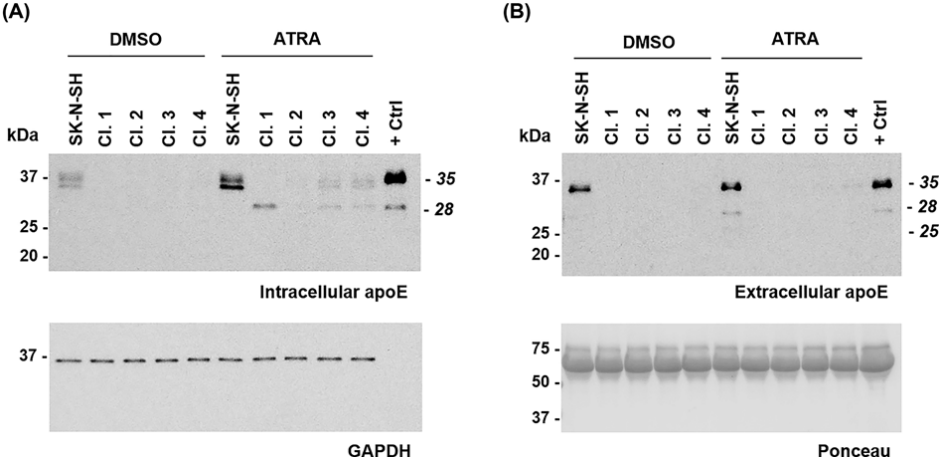
Selected clones show lower apoE expression than SK-N-SH cells SK-N-SH cells and selected clones 1–4 were cultured with 10 µM ATRA or DMSO control for 6 days. (**A**) Western blot for cell lysate showing intracellular apoE expression, and GAPDH expression used as a loading control. (**B**) Western blot showing extracellular apoE expression in the conditioned medium of the cells and Ponceau staining used as a loading control. SK-N-SH conditioned medium after 6 days of ATRA treatment was used as a positive control (+ Ctrl) in (A,B).

Western blots were performed to confirm the reduction in apoE expression in the cell lysate (intracellular apoE) and in the conditioned medium (extracellular apoE) in the selected clones (Figure 3). The parental SK-N-SH cells showed the 35-kDa full-length apoE, with higher expression after ATRA treatment than in the DMSO control condition. No expression was detected in DMSO conditions in any of the selected clones; however, whereas clone 2 showed no expression after ATRA treatment, clone 1 showed expression of a 28-kDa fragment and clones 3 and 4 showed low expression of both the 35-kDa and 28-kDa apoE (Figure 3A). ApoE fragmentation in SK-N-SH cells has previously been found only extracellularly in the cell medium [16]. However, this current data indicated the possibility of synthesis of a truncated 28-kDa apoE peptide that remains intracellular in some of the *APOE* KD clones. When analysing the conditioned medium after 6 days, the parental SK-N-SH cells showed full-length apoE in DMSO control conditions and full-length and 28-kDa and 25-kDa fragments after ATRA treatment, consistent with previous experiments. Importantly, none of the selected KD clones showed apoE expression under any condition (Figure 3B).

**Figure 4:**
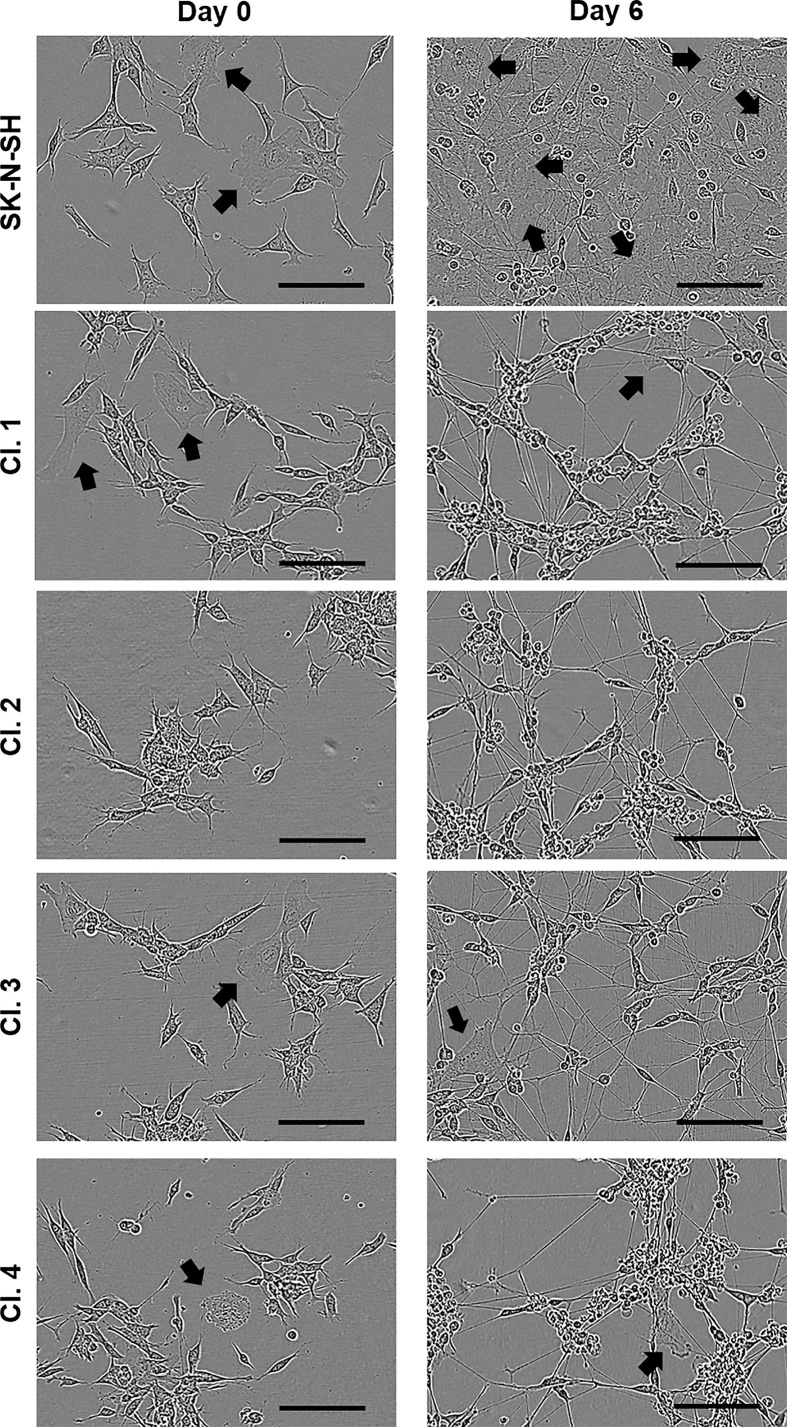
SK-N-SH and selected clones 1–4 show a change in morphology from days 0 to 6 of ATRA treatment Representative bright-field images of SK-N-SH and CRISPR clones at days 0 (before ATRA) and 6 of ATRA differentiation. Black arrows indicate substrate-adherent cells. Scale bar = 100 µm.

The SK-N-SH cells and the selected clones 1–4 showed a similar morphology before differentiation (Figure 4). After 6 days of ATRA treatment, the parental SK-N-SH cells showed two subpopulations of cells, the neuron-like cells (also known as N-type) and substrate-adherent cells (S-type, indicated by arrows in Figure 4), as previously described [19,20]. However, the four selected clones showed predominantly N-type cells based on the neuroblastic morphology found in culture, with only some S-type cells found in clones 1, 3 and 4 (Figure 4).

**Figure 5:**
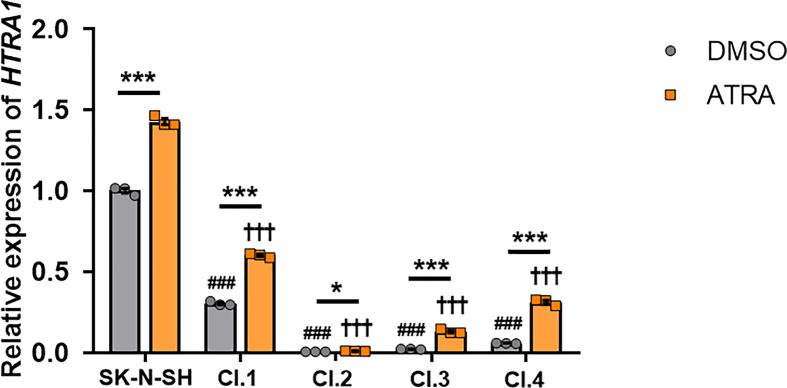
Selected clones show lower *HTRA1* expression than parental SK-N-SH cells SK-N-SH cells and selected clones 1–4 were cultured with 10 µM ATRA or DMSO control for 6 days. The mRNA expression of *HTRA1* was normalised to the housekeeper genes, *GAPDH* and *HPRT1*, and SK-N-SH DMSO expression was used as 1 for reference. Data are derived from one representative experiment performed in triplicate. Individual data points are shown. Histogram bars represent mean values and error bars represent S.E.M. Graph shows *P<0.05 and ***P<0.001, compared with each DMSO condition by two-tailed *t* test; ^###^*P*<0.001 compared with the SK-N-SH DMSO condition and ^†††^*P*<0.001 compared with the SK-N-SH ATRA condition by one-way ANOVA with Dunnett’s *post-hoc* analysis.

HtrA1 is a serine protease responsible for the generation of the 25-kDa apoE fragment [16]. *HTRA1* levels were analysed in parental SK-N-SH cells and the selected CRISPR clones after 6 days of ATRA treatment or DMSO control (Figure 5) by qPCR. Whereas the parental SK-N-SH cells and the CRISPR clones showed an increase in *HTRA1* expression when ATRA treatment was compared with DMSO control, there was a significant reduction in *HTRA1* expression in the *APOE* KD clones when compared with the parental SK-N-SH cells (Figure 5). Moreover, changes in *HTRA1* expression levels followed the same general pattern as observed for *APOE* expression (Figure 2).

**Figure 6:**
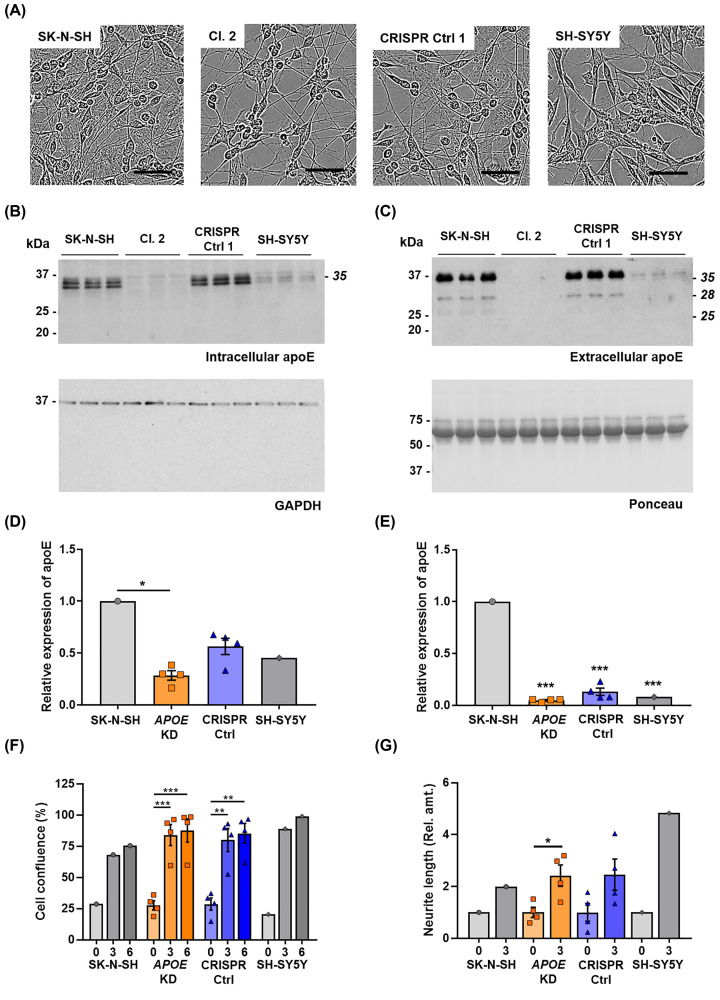
ApoE KD clone 2 and SH-SY5Y cells show lower apoE expression than SK-N-SH cells and CRISPR control clone 1 SK-N-SH, SH-SY5Y, four *APOE* KD clones and four CRISPR control clones were cultured with 10 µM ATRA for 6 days. (**A**) Representative brightfield images at 6 days of ATRA differentiation. Scale bars = 50 µm. (**B**) Representative western blot showing intracellular apoE expression and GAPDH expression as loading control in SK-N-SH, *APOE* KD clone 2, CRISPR control clone 1 and SH-SY5Y cells cultured with 10 µM ATRA for 6 days. (**C**) Western blot showing extracellular apoE expression in the conditioned medium and Ponceau staining used as a loading control. (**D**) Quantification of intracellular and **(E)** extracellular apoE expression in SK-N-SH cells, four *APOE* KD clones, four CRISPR control clones and SH-SY5Y cells. (**F**) Cell confluence measured over 6 days of ATRA treatment. (**G**) Neurite length measured at days 0 and 3 of ATRA treatment and normalised to day 0 of each experiment. Neurite length could not be analysed at day 6 as the confluence of the samples was over 90%. Data in (D,E) are normalised to SK-N-SH. Histogram bars in (D–G) represent mean values and error bars represent S.E.M, *n*=4, whereby individual data points represent different clones as biological replicates. Graph shows *P<0.05, **P<0.01, ***P<0.001, compared with SK-N-SH cells by one-way ANOVA with Tukey’s *post-hoc* analysis in (D–F), and two-tailed *t* test in (G).

To assess if the reduction in apoE expression in the CRISPR clones was due to a knock-down in the *APOE* gene or to the difference in cell phenotype, a new ATRA differentiation experiment was performed. The parental SK-N-SH cells, clone 2 and another 3 clones with double peak in the *APOE* sequence (clones 5, 6 and 7) were differentiated along with 4 CRISPR control clones (CRISPR control 1, 2, 3 and 4) that had undergone the CRISPR process and the monoclonal selection but had no alteration in the *APOE* sequence (Figure 1C, CRISPR/Cas9 edited cells), and the SH-SY5Y neuroblastoma cells, a neuroblastic subclone of the SK-N-SH cells. After 6 days of ATRA differentiation, KD clone 2 (representative of *APOE* KD clones) and SH-SY5Y cells showed only neuroblastic morphology, whereas SK-N-SH and CRISPR control clone 1 (representative of CRISPR control clones) showed a combination of neuroblastic and substrate-adherent cells (Figure 6A). ApoE expression was analysed by western blot in the cell lysate (intracellular apoE) and conditioned medium (extracellular apoE) (Figure 6B–E). As shown in the representative western blot, both SK-N-SH and CRISPR control clone 1 showed full-length apoE expression in the lysate and full-length and 28-kDa and 25-kDa fragment expression in the conditioned medium after ATRA treatment (Figure 6B,C). However, clone 2 and SH-SY5Y showed a reduction in apoE expression in both cell lysate and conditioned medium after ATRA treatment (Figure 6B,C). Western blot quantification of 4 *APOE* KD clones (Clone 2, 5, 6 and 7) and 4 CRISPR control clones was performed as shown in Figure 6D,E. The *APOE* KD clones showed a significant reduction in intracellular apoE when compared with the parental SK-N-SH cells; however, as expected, the CRISPR control clones did not show a significant reduction (Figure 6D). Moreover, in the conditioned medium after ATRA treatment, there was a significant apoE reduction in the *APOE* KD clones, the CRISPR control clones and the SH-SY5Y cells when compared with the SK-N-SH cells (Figure 6E).

Cell confluence and neurite length were also analysed and compared with each clone at day 0. Significant differences in confluency between lines were not detected over the 6 days of ATRA treatment (Figure 6F). Similarly, when neurite length was analysed at day 3, there was no significant difference between SK-N-SH cells, SH-SY5Y cells, *APOE* KD clones and CRISPR control clones (Figure 6).

## Discussion

The SK-N-SH neuroblastoma cell line (*APOE ɛ*3/3) has been published as a cell line to study the fragmentation of apoE [5], and the knock-down of *APOE* in this cell line thus provides a cell model to understand the role of apoE. CRISPR/Cas9 technology has revolutionised genome-editing over the last decade. Compared with previous genome-editing techniques such as zinc finger nucleases or transcription activator-like effector nucleases (TALENs), CRISPR is considered to be a simpler and more flexible method with a high editing efficiency [27]. The use of a specific gRNA to perform the editing in the CRISPR/Cas9 system was chosen to promote a high specificity of the target region. However, off-target effects have been described previously in the system as reviewed in [28]. The predicted off-target sites for the selected gRNA with a higher score was *TMEM175* obtaining 1.43 and had 4 mismatches in the sequence, that compared with *APOE* gene having a score of 100 and no mismatches indicates high specificity to bind the *APOE* gene over other sequences of the genome [25]. Four of the CRISPR clones that showed double peaks in the electropherogram were analysed for a knock-down in *APOE* expression. The four selected clones showed a reduction in *APOE* expression after ATRA differentiation, and the KD was confirmed by a reduction in protein level (Figures 2 and 3).

Previous studies have modified the expression of apoE in mouse models (as reviewed by Kim et al. (2014) [29]). Some of these studies in *APOE*-deficient mice showed that a lack of apoE results in synaptic alterations during aging in the neocortex and the limbic system [30], learning deficits [31] or cholinergic impairment and memory deficits [32]. Expression of apoE3, but not apoE4, in neurons of *APOE* KO mice restored protection against neurodegeneration [33]. One of the specific functions of apoE in the brain is thought to be to increase neurite outgrowth in an isoform-specific manner [34–37]. Moreover, in our previous study we demonstrated that the apoE 25 kDa N-terminal fragment also increased neuritogenesis in SH-SY5Y cells [16]. In this study we showed that the lack of apoE did not appear to affect proliferation (Figure 6F), and there was no significant difference in neurite outgrowth in the clones that lacked apoE compared with the CRISPR Control clones or SK-N-SH cells (Figure 6G). One explanation for this could be the difference in the cell cultures between the parental SK-N-SH and the CRISPR clones after ATRA treatment. There are two subpopulations of cells in the SK-N-SH cell line: N-type and S-type, and some reports suggest that they can transdifferentiate [19]. Based on our study, clone 2 that did not contain substrate-adherent cells (S-type cells) showed the lowest *APOE* expression. Moreover, the control clones that were manually selected after CRISPR but did not generate a DSB showed higher apoE levels than clone 2 but lower than the parental SK-N-SH cells. The CRISPR control clones showed predominantly N-type cells, but some S-type cells were found in culture. SH-SY5Y cells, characterised as neuroblastic, showed apoE expression levels that were as low as the SK-N-SH CRISPR clone 2. Based on our results, it therefore appears that apoE could be expressed by S-type cells that show a more epithelial phenotype and not the neuroblastic cells as also shown in Muñoz et al. (2018) [16]. Thus, we hypothesise that there are differences in expression mediated by the presence of distinct cell types in SK-N-SH cell populations and caution should be applied when considering experimental interpretation or the choice of these cells as a model for neuroscience applications. Besides this, the presence of the S-type cells on the SK-N-SH and the CRISPR control cultures might interfere with neurite quantification when comparing them with the N-type cultures in the CRISPR KD clones and SH-SY5Y cells (Figure 6A). Therefore, one of the main considerations of the study lies in the different populations of the neuroblastoma cell lines. Thus further experiments are required to identify whether apoE affects transdifferentiation of N-type and S-type cells.

The presence of the *APOE ɛ*4 allele is the major genetic risk factor of AD [1,38] and apoE is proteolytically cleaved in the brain in an isoform-dependent manner [4]. However, there is controversy in the literature regarding the enzyme responsible for apoE fragmentation and the specific function of the apoE fragments. The expression of apoE and the production of apoE fragments increases over time with ATRA treatment [16]. However, the *APOE* knock-down clones generated here showed a reduction in *HTRA1* expression concomitant with a reduction in *APOE* expression (Figures 2 and 5). Under normal conditions, neurons do not express apoE, however some studies have shown that apoE expression is up-regulated upon excitotoxic stress induced by kainic acid [39,40]. Moreover, astrocytes and astrocytic cytokines, such as interleukin-1β, have been shown to activate apoE expression in neurons, mediated through the Erk pathway [41,42]. Additionally, HtrA1 is known to be expressed in the brain during development in mice, controlling neuronal maturation and developmental survival [43,44] and HtrA1 expression is up-regulated in response to elevated tau protein concentrations [45]. One possibility is that, under normal circumstances, neuronal cells (as modelled by the N-type in the SK-N-SH cells, such as clone 2 in the present study or SH-SY5Y cells) show low or no levels of HtrA1. However, when apoE is present, there is an up-regulation in HtrA1 expression, and therefore in apoE 25. It has been shown that apoE plays a role in neuroprotection [41,46], and it remains to be determined if apoE 25 has a different role under stress conditions. Further studies are required to understand the link between HtrA1 and apoE expression and the loss of neuroprotection when apoE levels are reduced or absent; the *APOE* knock-down clones generated in this study can be used for such studies.

In conclusion, we used CRISPR/Cas9 technology to generate different clones of SK-N-SH cell lines with *APOE* KD expression that can be used as models to study apoE function, including fragmentation by exogenous addition of apoE isoforms and the interaction between apoE and HtrA1.

## Data Availability

Data are available on request.

## Abbreviations

AD: Alzheimer’s disease
apoE: apolipoprotein E
apoE 25: apoE 25-kDa N-terminal fragment
ATCC: American Type Culture Collection
ATRA: all-*trans* retinoic acid
cDNA: complementary DNA
DMEM: Dulbecco’s modified Eagle’s medium
DSB: double-strand break
EDTA: ethylenediaminetetraacetic acid
FBS: foetal bovine serum
gRNA: guide RNA
HRP: horse radish peroxidase
HtrA1: high temperature-requirement serine protease A1
KD: knock-down
PAM: protospacer adjacent motif
PBS: phosphate buffer saline
PBS-T: 1× PBS-0.1% (v/v) Tween 20
qPCR: quantitative PCR
